# Implementation of the WHO “Safe Surgery Saves Lives” checklist in a podiatric surgery unit in Spain: a single-center retrospective observational study

**DOI:** 10.1186/s13037-015-0075-4

**Published:** 2015-08-28

**Authors:** Jaime García-París, Manuel Coheña-Jiménez, Pedro Montaño-Jiménez, Antonio Córdoba-Fernández

**Affiliations:** Departament of Podiatry, University of Seville, Avicena Street, 41009 Seville, Spain

## Abstract

**Background:**

The Surgical Safety Checklist (SSC) is a tool developed by the World Health Alliance for Patient Safety, to assist health professionals in improving patient safety during surgery. Numerous specialties have incorporated this into their clinical practice. The purpose of this study is to adapt and implement this tool within the field of podiatric surgery and to evaluate its impact upon safety standards and post-surgical complications.

**Methods:**

An analytical, observational, longitudinal study has been performed retrospectively. The implementation of the Surgical Safety Checklist in podiatric surgery took place over a 10-month period. The sample is made up from the medical histories of patients who were operated on (n = 134) in the University of Seville’s podiatric clinic. The sample was divided into three groups: those prior to the implementation process (65 subjects), those after the implementation process: without the SSC (35 subjects) and those with the SSC (34 subjects). The safety standards included in the tool were analysed in conjunction with the results and post-operative complications.

**Results:**

An improvement was seen in compliance with the Prophylaxis Protocol and the correct completion of the Informed Consent (p = 0.00), as well as a statistically significant relationship between the correct use of antibiotic prophylaxis and the use of the Surgical Safety Checklist (p = 0.049). The results demonstrate a reduction in the number of post-operative days (p = 0.012). No cases of surgery being performed in the wrong place were found in this study.

**Conclusions:**

The Surgical Safety Checklist allows us to improve compliance with the safety protocols recommended by the scientific community, and consequently to reduce the incidence of complications related to surgery and to improve patient safety during elective podiatric surgery.

## Background

Patient Safety has been discussed since the Aristotelian principle “primum non nocere” but it is still highly relevant today and has gained strength since the creation of the World Alliance for Patient Safety [1].

Finding the cause of adverse events in healthcare and a means of reducing their occurrence is a cause for concern for healthcare professionals and managers. The first publication that highlighted healthcare-related adverse events, and as such, sparked interest in offering safer healthcare and correctly identifying any adverse events in the sector was the Institute of Medicine’s (IOM) 1999 publication ‘To err is human: building a safer health system’ [2]. This estimated that between 44,000 and 98,000 people die every year as a result of medical errors in the United States.

Other studies suggest that the incidence rate is between 2.9 and 16.6 [3–6]. The highest incidence of adverse events is registered in surgical specialities [7].

Given these figures, the World Alliance for Patient Safety outlines specific bi-annual goals. The Surgical Safety Checklist (SSC) has been developed from two projects which they have carried out: “Clean Care is Safer Care” [8] and “Safe Surgery Saves Lives” [1]. It is an easy-to-use, measurable set of safety checks, adaptable to different healthcare settings.

It is well-known that fatigue, stress and the development of complex procedures reduces the precision and speed of the human memory [9, 10]. These studies demonstrate the utility of checklists as a safe and useful tool to help minimise human error.

De Vries [11] introduced a checklist encompassing a patient’s complete medical history. This was later adapted by other authors, including Boscá et al. [12] for use in interventional radiology, and Perea et al. [13] to dental surgery.

There are few studies related to patient safety in the field of podiatry. Jones y Levy (2012) [14] refer to the need to improve the educational model for podiatrists in terms of patient safety and in regards to error disclosure to improve professional development. Other publications in the field of podiatry address some patient safety standards, such as those related to antibiotic prophylaxis [15, 16], the incidence of thrombosis-embolism [17, 18], the surgical preparation of the skin [19–21] and the prevention of surgery in the wrong site [22, 23]. To date, the majority of the bibliography refers to isolated cases or short series on which empirical evaluations have been performed. Coheña et al. [24] are pioneers in this issue in podiatry, having proposed an adapted version of the SSC for podiatric surgery, without results.

The purpose of this study is to evaluate the impact of the SSC proposed by Coheña et al. in regards to safety standard compliance and the reduction of surgical complications in podiatric surgery.

## Methods

### Setting

Based on the SSC implementation guide in the Safe Surgery Saves Lives [1] programme, in order to implement the SSC in podiatric surgery there are 10 phases, these are identified in the Gantt chart, where each activity is recorded together with the time required for their implementation, (Table 1 Implementation phases). This process took ten months and took place in the Podiatric Clinic at the University of Seville (ACP).

**Table 1:** Implementation phases

Phases	Process
1. Need for implementation and creation of a working group	- Identification of the problem and precision of the verification checklist as a solution
	- Creation of a team that will develop the implementation.
2. Definition of purpose of the checklist and the bibliographic review	- Identification of the people to whom the checklist is performed and the type of activity to which this tool is aim to be related to.
3. Analysis of the situation	- Observation of the context where the implementation will be developed.
	-Evaluation of the strengths and weaknesses.
4. Elaboration of an activity checklist	- Creation of a sequential list of the actions that are being performed and on which interventions are required.
5. Design of the verification checklist	- Creation of a preliminary format with the help of an activity list.
6. Revision of the checklist	- Periodic review of the checklist with members of the team and participants of the implementation.
7. Proof of the functionality of the verification checklist	- A small-scale evaluation of the checklist.
	- Training for professionals.
	- Analyses of the experience through the direct observation or questionnaires
8. Approval of the checklist	- Performs of the necessary modifications.
9. Training for professionals	- Training through workshops, talks, live simulations.
10. Regular re-assessment of the checklist	- Analyses of changes on the context of functioning
	- Performs of readjustments according to the changes in the situation.

The process of implementation lasts 10 months and it has 10 different phases

Around 150 surgical podiatric procedures are carried out at this centre on an out-patient basis, from nail surgery to osteoarticular surgery with orthopaedic fixation devices under local anaesthetic. As a new tool in the field of podiatry and as recommended by other authors [25–27], an intensive training programme was undertaken during the implementation process before any data was collected. This programme included the development of a handbook, briefings and practical workshops.

Evaluation focused on identifying changes that occurred in patients as a result of using the SSC, comparing the three groups which the sample had been divided into: the pre-implementation group/the group without the SSC and the group with the SSC.

A retrospective quantitative review was made from certain documents from the medical histories as an indicator of the level of compliance to the safety standards established by the WHO. Following the same protocol as this study, the researchers are Doctors in Podiatry and experts in management and quality of care.

### Study design

This analytical, observational and longitudinal study was retrospectively evaluated.

Simple random sampling was used and the sample size calculated using a formula where n is the sample size, N is the population size, Z is 0.05 y p is the reference variable.

$$ \mathrm{n} = \frac{N\times {Z}^2 \times p \times q}{d^2 \times \left(N-1\right)+{Z}^2\times p\times q} $$

**Fig. 1:**
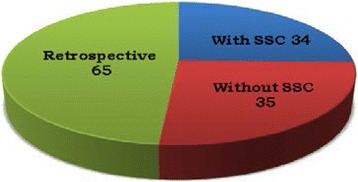
Distribution of study groups. Retrospective group 65 subjects; Without SSC group 35 subjects; With SSC group 34 subjects

The sample consisted of 134 patients, divided into the previously described groups. (Fig. 1 Distribution of study groups)

.

The main variable is the degree of patient safety during surgery, in relation to compliance with the SSC-defined safety standards. Independent variables are shown in Table 2. (Table 2 Definition of the independent variables).

**Table 2:** Description of the independent variables

Independent variables	Definition
Surgeon	Professional that performs the surgery.
Sociodemographic variable	This includes the age and the gender.
American Society of Anesthesiologists (ASA)	The surgical risk that a patient can experiment according to the measuring scale of the American Society of Anesthesiologists. The ordinal scale from ASA I to ASA V.
Type of surgery	Osteoarticular surgeries with or without implants and nails or skin surgery.
Fulfillment of the Informed Consent	It measures the correct fulfillment of the informed consent, codified in complete, incomplete or nonexistent.
Identification of the surgical site	It measures the correct identification of the anatomical site where the surgical procedure is going to be performed in the medical history. When the identification is correct, it is codified with a YES, or NO when it is incorrect. The reasons of a NO codification can be an inconsistency of the identification of the surgical site between the documents, or the anatomical site of the operation is not identified, or a surgery has been performed in the wrong site.
Fulfillment of the DVT Prophylaxis Protocol (DVTPP)	This is applied to patients undergoing surgery and assesses the risk of a thromboembolism. On the other hand, it measures the level of compliance of the protocol. A “Secure” codification is given to the patient when the assessment page of DVTPP risk is completed, when a DVT prophylaxis is required or when the assessment is completed and the patient does not require it or prophylaxis is not established as a treatment. The rest of the variations are considered insecure practices.
Correct use of the antibiotic prophylaxis	Antibiotic prophylaxis is require when the patient presents 3 or more risk factors (≥65 years, Diabetes Mellitus, malnutrition, obesity, ASA ≥ 3, smoking habits, coexistence of the infection in other locations, immunosuppression and radiotherapy treatment) in the cases of surgery with osteosynthesis materials. It is considered a secure practice when the subjects require antibiotic prophylaxis and it is established as a treatment; or when, on the contrary, the antibiotic prophylaxis is not required or established.
Infection of the surgical site	This happens when clear signs of infection are described in the medical history (such as pain, swelling, suppuration, erythema, redness) or when a local or oral antibiotic is prescribed during the postsurgical process.
Postoperative days	From the days of the operation till the date of discharge.

## Results

The average age of the sample group was 47.49 years old, with a standard deviation of 22.124. In terms of gender, 73.9 % were female and 26.1 % male. In regards to the Surgical Risk Calculation, 51.5 % of patients were classed as ASA I, 47 % were classed as ASA 2 and only 1.5 % as ASA 3.

In terms of the type of surgery carried out, the highest percentage involved nail/skin surgery (66.4 %), followed by osteoarticular surgeries with implants (23.1 %) and osteoarticular surgeries without implants (10.4 %).

### Correct compliance with the deep venous thromboembolic prophylaxis protocol (DVTPP)

Through the use of Pearson’s Chi-square Test, p = 0 (>0.05) a significant relationship is observed between the “WITH checklist group” and the correct practice of DVTPP. (Table 3A-B Comparison chart: Correct compliance with the DVTPP risk assessment and Chi-square test). The protocol was proposed by Autar R. [28] and was incorporated into podiatric surgical care at the ACP. The results of this study demonstrate that the SSC helps to improve compliance with the DVTPP. Truran [29], in his pre-post SSC implementation study, compares the compliance rates with the DVTPP, noting that non-compliance fell from 6.9 % to 2.1 %. This study found that the non-compliance rate was 68 % in the period prior to the implementation of the SSC, a figure that decreased to 24 % in the without SSC group and to 8 % in the with SSC group. It could be argued that this difference is due to the increased awareness of patient safety after the implementation period. The high levels of non-compliance found during this study in comparison to that of other studies could be explained by a failure to adhere to the protocol. This is likely because cases of thromboembolic complications in podiatric surgery are much fewer than in general surgery where these types of complications are common and stricter protocols exist.

**Table 3:** Comparison chart Correct Fulfillment of the DVTPP risk assessment

A.				
			DVTPP Assessment	
			Secure	Insecure
Types of data	With SSC	Recount	28	6
		% in the DVTPP security	47.5 %	8.0 %
		Revised residues	5.2	−5.2
	Without SSC	Recoaunt	17	18
		% in the DVTPP security	28.8 %	24.0 %
		Revised residues	.6	-.6
	Retrospective	Recount	14	51
		% in the DVTPP security	23.7 %	68.0&
		Revised residues	-5.1	5.1
B. Chi-square test				
		Value	gl	Sig. Asymptotic (bilateral)
Pearson chi-square		33.898	2	.000
Number of valid cases		134		

A significant relation has been observed between the group WITH checklist and the secure practice of the DVTPP assessment (>0.05)

### Correct antibiotic prophylaxis pratice

Antibiotic prophylaxis is a controversial issue among health professionals, including podiatrists; nonetheless, the correct use of antibiotic prophylaxis reduces the risk of post-surgical complications, offering patients health benefits and an increased quality of care, as well as having financial repercussions [30].

This study makes use of the recommendations made by Córdoba et al. [15] and Mosquera et al. [16] in their reviews as a means of assessing the usefulness of antibiotic prophylaxis. The results of this study demonstrate a significant relationship between the use of the SSC and the correct usage of antibiotic prophylaxis (p = 0.049). (Table 4 Correlation between the use of the SSC and correct use of antibiotic prophylaxis). Similarly, other authors [31] also note a significant improvement in the correct usage of antibiotic prophylaxis (57 % in the period prior to the SSC and 77 % in the period post). Rydenfält [32] observed that the standard associated with antibiotic prophylaxis in the SSC was one of the easiest to comply with.

**Table 4:** Correlation between the use of SSC and correct use of the Antibiotic prophylaxis

A.						
			Antibiotic prophylaxis			
			Required, established treatment	Required, not established treatment	Not required, but established treatment	Not required and not established treatment
Types of data	With SSC	Recount	15	1	3	15
		% in the antibiotic prophylaxis	36,6 %	4,3 %	37,5 %	24,2 %
		Revised residues	2,0	−2,5	,8	-,3
	Without SSC	Recount	6	7	1	21
		% in the antibiotic prophylaxis	14,6 %	30,4 %	12,5 %	33,9 %
		Revised residues	−2,0	,5	-,9	1,9
	Retrospective	Recount	20	15	4	26
		% in the antibiotic prophylaxis	48,8 %	65,2 %	50,0 %	41,9 %
		Revised residues	,0	1,8	,1	−1,4
B. Chi-square test						
				Value	gl	Sig. Asymptotic (bilateral)
Pearson chi-square	12,646	6	.049			
Number of valid cases	134					

### Surgical site infection rate

De Vries [33] and Tillman [34] indicate that surgical site infection is the most frequent postsurgical complication and one with the highest impact upon the health/illness process of the patient, satisfaction levels and healthcare spending. According to Butterworth [35] and Zgonis [36] an infection rate of between 0.5 and 6.5 % is accepted as normal in elective foot-ankle surgery amongst podiatric surgeons. This study found a much higher total surgical site infection rate (15.3 %) than that accepted as normal by these authors. This can be explained by the teaching nature of the centre where this study was undertaken and the inherent bias of the medical histories involved. In the Table 5 (Table 5 Comparative analyses of data on surgical site infection) shows the comparative figures between this study and the research of Bliss [37], Tillman [34] and Haynes [38]. A reduction in the surgical site infection rates between the different groups are observed, reflecting lower infection rates in the groups where the SSC was used.

**Table 5:** Comparative analyses of data about infection on the surgical site

Authors	Retrospective	Without SSC	With SSC
Present Study	9.2 %	4.6 %	1.5 %
Bliss et al. [42]	3.4 %	2.8 %	1.4 %
Tillman et al. [39]	1.7 %	-	0.7 %
Haynes et al. [43]	6.2 %	-	3.4 %

Furthermore, a significant relationship is observed between the reduction in surgical site infection rate and antibiotic prophylaxis (p = 0.019) (Table 6 A-B Relationship between surgical site infection and the correct usage of antibiotic prophylaxis). This is something which leads us to believe that an indirect correlation exists between the use of the SSC and the reduction in the surgical site infection rate.

**Table 6:** Relationship between Surgical site infecion and the secure use of the antibiotic prophylaxis

A.					
			Antibiotic prophylaxis security		
			Secure	Insecure	
Surgical site Infection	Yes	Recount	11	9	
		% in the Antibiotic prophylaxis security	11,0 %	29,0 %	
		Revised residues	−2,4	2,4	
	No	Recount	89	22	
		% in the Antibiotic prophylaxis security	89,0 %	71,0 %	
		Revised residues	2,4	−2,4	
B. Chi-square Pearson test and Fisher’s exact stadistical test					
	Value	gl	Sig. Asymptotic (bilateral)	Sig. Exact (bilateral)	Sig. Exact (Unilateral)
Pearson chi-square	5.948	1	.015		
Fisher’s exact statistical test				.022	.019
Number of valid cases	134				

(When it use the antibiotic prophylaxis security correctment, the surgical site infection decrease to stadistical significative way)

### Correct completion of informed consent

Numerous authors [39, 40] highlight the importance of patient-surgeon communication and consider the inclusion of the patient in their treatment the fundamental premise of healthcare. The informed consent form is a scientifically endorsed tool available to evaluate this relationship. Yet, in clinical practice is not always employed correctly, impacting upon communication, safety and affording the patient a grounds for claim [41].

The results of this study demonstrate a connection between the use of the SSC and higher levels of compliance and completion of the informed consent in the surgical process. De Vries [42] analysed 294 complaints made to Dutch health professionals, he indicated that 100 % of the 23 complaints registered in relation to the informed consent could have been avoided through the use of the SSC. Indeed, Cavallini [43], through incorporating the SSC into the quality programme at the centre where his study was undertaken, increase the informed consent completion rate to 99.76 %.

### Number of post-operative days

**Fig. 2:**
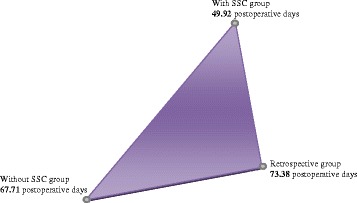
Comparative graphic on the number of postsurgical days. A 49.92 with SSC in comparison with 67.71 without SSC and 73.38 Retrospective

The Kruskal-Wallis test for independent samples was used to associate the “post-operative days” independent variable to the study’s different groups, establishing a significance level of 0.05. This study found a significant relationship between the use of the SSC and the reduction of post-operative days, as is shown in Fig. 2 (Fig. 2 Comparative graphic on the number of postsurgical days) and Table 7 (Table 7 Statistical data on number of postoperative days).

**Table 7:** Statistical data to number of postoperative days

	Statistical test	Standard error	Deviation Statistic test	Sign	Adjoining sign
With SSC-Retrospective	−23.459	8.129	−2.886	.004	.012

The use of SSC decrease the postoperative days statistically significant (0,012)

This result confirms that the SSC affords the surgical team a visual and verbal reminder of the recommended safety measures, thereby reducing reliance on memory and improving compliance with basic safety standards [32, 39–43], consequently reducing the post-surgical period.

A retrospective analysis of medical histories was used in this study. The quality of the data collected was dependent upon the quality of the documentation in the medical and legal records. Given that compiled data may not directly reflect clinical practice, and therefore, as Panesar et al. [44] suggests, an infra-supra register might exist, Soria-Aledo [45] acknowledged this as a limitation in their studies. This study was able to minimise the Hawthorne effect by dividing the sample into three groups, as has been described by various authors [29, 46].

## Conclusions

Just as the attitude and motivation of professionals can change, so can clinical practice. It is therefore necessary to establish a monitoring process for the SSC, performing audits and re-editing where necessary in order to make it more efficient and effective for professionals. Significant improvements have been seen in the utilization of patient safety protocols such as the DVTPP and antibiotic prophylaxis, as well as a reduction in post-operative days. These changes improve, both directly and indirectly overall patient safety, reducing surgical complications such as surgical site infections. After analysing all of the tests used to evaluate the SSC implementation process in podiatric surgery, we believe the study’s objectives have been fulfilled and confirm that the SSC is a useful and effective tool in the improvement of patient safety. We believe that further studies, over longer timeframes and in other podiatric surgical centres are necessary in order to gather further scientific evidence.

### Ethical considerations

This investigation project meets with the approval and acceptance of the Ethical Committee of Experimenting of the University of Seville to investigate with human subjects and it adjusts to the current regulations in Spain and the European Union. To finish, informed consent was obtained of each participant after being completely informed before their participation in this study.

## Abbreviations

WHO: World Health Organization
SSC: Surgical Safety Checklist
ASA: American Society of Anesthesiologists
IC: Informed Consent
DVTPP: Deep Venus Tromboembolic Prophylaxis Protocol
